# Engineering solutions for a better world

**DOI:** 10.1038/s44172-022-00007-6

**Published:** 2022-05-26

## Abstract

Today we proudly launch *Communications* *Engineering*, a selective multidisciplinary journal which brings together the entire engineering community.

C*ommunications* *Engineering* is a journal from the *Nature* Portfolio dedicated to publishing research across all engineering fields. Our publications report an advance in thinking for a field which has strong potential to open up new engineering opportunity, capability or benefit. Engineering research in its broadest sense uses scientific principles to investigate a problem, and subsequently develop, implement, maintain and enhance solutions. We equally accept applied science contributions which create new and valuable insight towards solving engineering problems. Our key goals for the journal are threefold. First, we want to publish inspirational research, reviews and commentary which have clear impact for a focused community. Second, we want to provide a high quality author experience. Finally, we want to be a strong supporter of a diverse engineering workforce.

Our first research content published today shows how our multidisciplinary scope can bring together the engineering community in several different ways.

 We are living through difficult times. A global pandemic, the climate crisis and war have all led to increased insecurity in accessing food, water, energy, shelter, good health and work. The United Nations Sustainable Development Goals (SDGs) provide a framework within which to define targets to achieve a more equitable and sustainable world. We believe that by bringing together engineering communities which are thinking in different ways about the SDGs in one platform we can create a strong sense of a shared mission in addressing these grand challenges. Today we begin by publishing two papers in the field of sustainable transport. One reports an approach for upcycling of end-of-life vehicle waste (See Wyss et al.). The other contributes insights towards understanding automotive lithium ion battery degradation (see Monroe et al.). From resilient infrastructure to water purification, digital technologies and more, we look forward to publishing and curating a wide array of topical content addressing the SDGs.

We are delighted that the submissions we have received thus far have come from a huge range of engineering fields. *Communications* *Engineering* is part of the *Nature* Portfolio, which has already established itself in energy, electronics, artificial intelligence and biomedical engineering amongst others. *Communications* *Engineering* emphatically supports these important directions. However, we also warmly welcome contributions from researchers in fields less familiar with the Nature Portfolio, chemical, civil, structural and environmental engineering, logistics, networks and transport amongst others, another benefit of our broad scope.

As examples, in this first release, we present two papers exploring approaches to the management and protection of space-based technologies. Bartels and colleagues describe an approach to allow for precision monitoring of space objects’ orbits to prevent collisions and debris. Meanwhile Polese and colleagues report an approach for future high capacity 6G telecommunications systems to share electromagnetic spectrum frequencies above 100 GHz with sensitive satellite global monitoring technologies.

Our broad scope also allows us to be free to explore research which crosses disciplinary boundaries. Finding a home for interdisciplinary papers can be difficult. Today we offer two papers which explore mechanical engineering in biomedical contexts. Mouthuy and colleagues report the application of robotic technologies to bioreactors for tissue engineering. Hofstetter and colleagues describe the development of motors without magnets which can allow robotic actuation inside a magnetic resonance imaging machine during imaging.

Our service to our contributors is as important to us as the content we publish. *Communications* *Engineering* has both an internal team of professional editors and an external editorial board made up of active academic researchers from across a breadth of fields. The editorial structure and collaborative culture at *Communications* *Engineering* is designed to offer a powerful combination of specialist expertise and editorial drive to provide efficient and high quality editorial and technical peer review. Good quality and timely peer review improves the impact of the research we publish. We also strive to maximise impact by publishing all our content open access, by offering transparent peer review and by encouraging sharing of all data and code which has contributed to our publications.

Finally it is our firm commitment in the months and years ahead to showcase and provide opportunities for those from groups historically excluded from engineering and engineering research careers. For example, the gender ratio of university graduates in engineering across many countries is consistently biased in favour of men^1^. And while female representation is increasing at the assistant and associate professorial levels, there is much to be done to ensure this propagates to the highest level in academia. Recent data from the UK and USA show that only around 13.5% of full time professors across all engineering sectors were female^2,3^. Increasing the numbers of women opting to choose engineering as a career is one approach to addressing the skills shortage in this sector and ensure there are enough workers to fill the jobs of tomorrow.

The publication of the first content is a key moment in the life of a new journal. We are hugely grateful to all those who have supported the journal up to this point. We are excited to join the engineering community, to support promising new ideas and propel research towards real world utility for the benefit of society and a better world.
